# Euglycemic Diabetic Ketoacidosis Presenting as Hypoglycemia in a Patient With Type 2 Diabetes and Von Gierke’s Disease

**DOI:** 10.7759/cureus.52104

**Published:** 2024-01-11

**Authors:** Emmanuel Tito, Akshaya Ramaswami, Ron Milbocker, Darwin Edmond

**Affiliations:** 1 Internal Medicine, Western Michigan University Homer Stryker M.D. School of Medicine, Kalamazoo, USA; 2 Medicine and Surgery, Western Michigan University Homer Stryker M.D. School of Medicine, Kalamazoo, USA

**Keywords:** empaglifozin, hypoglycemia, von gierke disease, diabetic ketoacidosis (dka), diabetes mellitus

## Abstract

Von Gierke’s disease (VGD) is rarely associated with type 2 diabetes mellitus (DM2). We present a case of VGD with DM2 that presented with hypoglycemia in the setting of diabetic ketoacidosis (DKA) in a young male with VGD using the sodium-glucose co-transporter 2 (SGLT2) inhibitor empagliflozin. The patient required resuscitation with fluids containing glucose prior to the administration of insulin for DKA protocol with significant clinical improvement. This case demonstrates a rare presentation of DKA with hypoglycemia.

## Introduction

Von Gierke’s disease (VGD), also known as glycogen storage disease type 1 (GSD1), is an inherited metabolic disorder caused by a deficiency of glucose-6-phosphatase (G6Pase) that cleaves glycogen into glucose, thus allowing glycogenolysis and gluconeogenesis. It comprises two major subtypes, GSD1a and GSD1b. In GSD1a, there is a deficiency of the enzyme G6Pase, and GSD1b results from mutations in the SLC37A4 gene on chromosome 11q23.3 [1]. GSD1a leads to hypoglycemia and lactic acidosis in the event of fasting. Sodium-glucose co-transporter 2 (SGLT2) inhibitors are associated with euglycemic diabetic ketoacidosis (DKA) due to increased urinary excretion of glucose, causing glucose depletion and dehydration [2]. The combination of fasting and urinary excretion of blood glucose can result in significant fasting hypoglycemia in individuals with VGD. This case is a unique presentation of euglycemic DKA masqueraded by low serum glucose in a patient with GSD1a and type 2 diabetes mellitus (DM2) on an SGLT-2 inhibitor.

## Case presentation

The patient is a 36-year-old male with a past medical history of VGD, DM2 on the SGLT2 inhibitor empagliflozin, hepatocellular adenoma status post partial hepatectomy, obesity, and obstructive sleep apnea who presented to the hospital with nausea and right-sided abdominal pain that woke him up from sleep. The patient reported that he felt like his “liver was bouncing” and that the pain was significantly worse than his chronic right upper quadrant pain. He had a decreased appetite, resulting in him missing two meals. He also reported an episode of non-bloody, non-bilious vomiting, after which his mother measured his blood glucose at 30 mg/dL, prompting his presentation to the hospital emergency department. He denied cough, shortness of breath, chest pain, diarrhea, constipation, or changes in urination in the days leading up to admission.

In the emergency department, the patient had a maximum heart rate of 114 beats/min, blood pressure of 136/65 mmHg, a temperature of 37.1°C, and oxygen saturation of 97% in ambient air. Examination findings were remarkable for minimal right upper quadrant tenderness and hepatomegaly. Cardiovascular, respiratory, and neurological exams were unremarkable.

At admission, laboratory studies showed mild neutrophilic leukocytosis (white cell count: 12.6 × 10^9^/L, reference range: 4.0-11.0 × 10^9^/L), macrocytic anemia (hemoglobin: 13.1 g/dL, reference range: 13.5-17.5 g/dL), normal platelet count, elevated aspartate aminotransferase (89 U/L, reference range: 0-37 U/L), elevated alanine aminotransferase (66 U/L, reference range: 6-37 U/L), and elevated blood alkaline phosphatase (378 U/L, reference range: 40-129 U/L). Total bilirubin was mildly elevated (1.9 mg/dL, reference range: 0-1.2 mg/dL). Serum glucose was extremely low (19 mg/dL, reference range: 70-99 mg/dL). Total protein, lipase, ammonia, potassium, sodium, vitamin B12, and folate were in the reference range.

**Figure 1:**
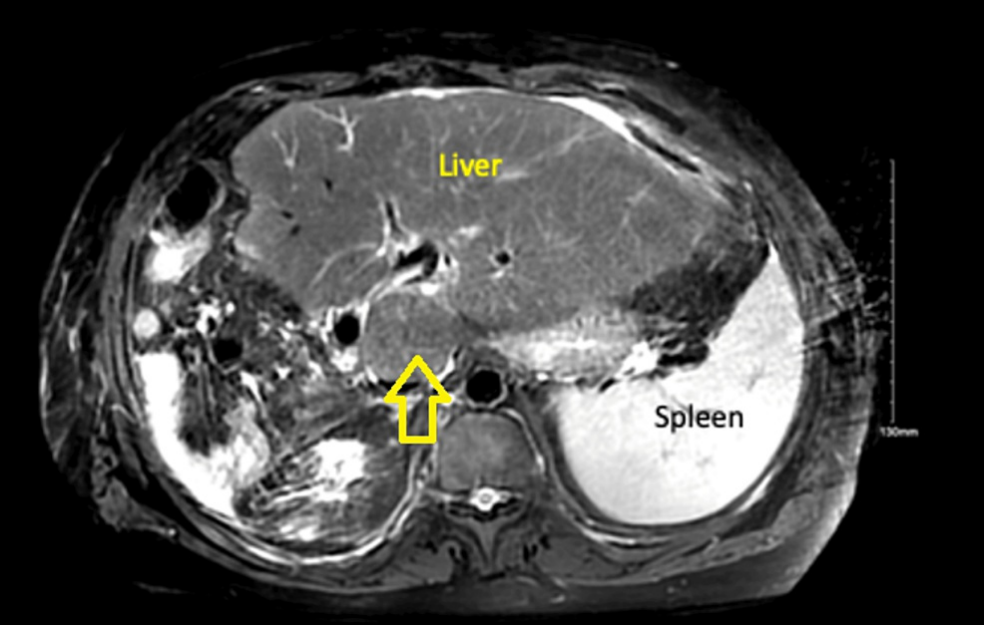
MRI abdomen with and without contrast showing compensatory hypertrophy of the left lobe of the liver with post-operative changes as a result of right hepatectomy and partial left lateral resection There are multiple arterially enhancing lesions representing hepatic adenomas (yellow arrow).

Additional studies revealed an elevated beta-hydroxybutyrate (4.96 mmol/L, reference range: 0.02-0.27 mmol/L), elevated lactate (17 mmol/L, reference range: 0.7-2.5 mmol/L), venous pH of 7.12 (reference range: 7.32-7.42), bicarbonate of 9 mmol/L (reference range: 23-32 mmol/L), and high anion gap of 35 (reference range: 9-18 mmol/L). Urinalysis showed ketonuria (80 mg/dL, reference range: negative), proteinuria (100 mg/dL, reference range: negative), and glucosuria (500 mg/dL, reference range: negative). Influenza A/B, respiratory syncytial virus (RSV), and COVID-19 by PCR were negative. Urine and blood cultures did not yield any bacterial growth. Right upper quadrant ultrasound showed mild splenomegaly, evidence of prior cholecystectomy, and no intrahepatic or extrahepatic biliary duct dilation. Computed tomography (CT) of the abdomen and pelvis did not show any acute findings. Magnetic resonance cholangiopancreatography (MRCP) showed postoperative changes related to right hepatectomy and left lateral partial resection. There were also multiple arterially hyper-enhancing lesions in the left hepatic lobe, the largest in hepatic segment 3 measuring up to 1.8 cm, not significantly changed when compared to prior examination (Figure 1). These were favored to represent small adenomas.

Based on the patient’s initial presentation, the differential diagnosis included DKA and acute biliary obstruction. Other potential explanations for the patient’s symptoms at the time of presentation included pancreatitis, acute liver infarct, ruptured hepatic adenoma, peptic ulcer disease, gastritis, and ischemic colitis, all being less likely given clinical history, minimal abdominal pain on the exam, and imaging results. Our patient presented with a point-of-care glucose of 12 mg/dL, thought to be precipitated by his glycogen storage disease in the setting of a history of partial hepatectomy. Low serum glucose level was consistent with point-of-care testing. The rest of his comprehensive metabolic panel and his elevated serum ketones and acidic venous pH established the definite diagnosis of SGLT2 inhibitor-induced euglycemic DKA with lactic acidosis presenting as hypoglycemia.

The patient was resuscitated with intravenous (IV) isotonic saline to correct his lactic acidosis. He was subsequently started on DKA protocol with an infusion at 0.1 units/kg/h. Dextrose 5% mixed with 0.45% normal saline infusion was placed to maintain the blood glucose goal between 140 and 180 mg/dL. The infusate was later switched to dextrose 10% with 0.45% normal saline infusion due to persistent hypoglycemia. Basic metabolic profile (BMP), magnesium, and phosphate were checked every four hours for electrolyte replacement. Once the anion gap closed, the patient was transitioned to long-acting and short-acting insulin based on a sliding scale. Empagliflozin was discontinued from the time of admission. Gastroenterology was consulted to perform an esophagogastroduodenoscopy (EGD) due to persistent vomiting at admission. EGD was unremarkable. Given his mild abdominal pain and recent abdominal surgery, a colonoscopy was performed, which revealed sigmoid diverticulosis and small internal hemorrhoids. Pathology from right and left colon biopsies showed focal active colitis (FAC). Diabetic education was provided while in the hospital. The patient was hospitalized for seven days and discharged home on insulin therapy. He was advised not to use empagliflozin at discharge. He was sent home with no neurologic sequelae. The patient was counseled about the risks, prevention, and treatment of hypoglycemia. Following treatment with insulin infusion and IV fluids, lactic acid and metabolic acidosis returned to normal. All his symptoms significantly improved at the time of discharge. He underwent physical and occupational therapy while in the hospital, gradually returning to his baseline activity. One week later, the patient followed up with his primary care provider and reported a complete resolution of symptoms. The patient did not have any neurological sequelae noted during follow-up.

## Discussion

**Figure 2:**
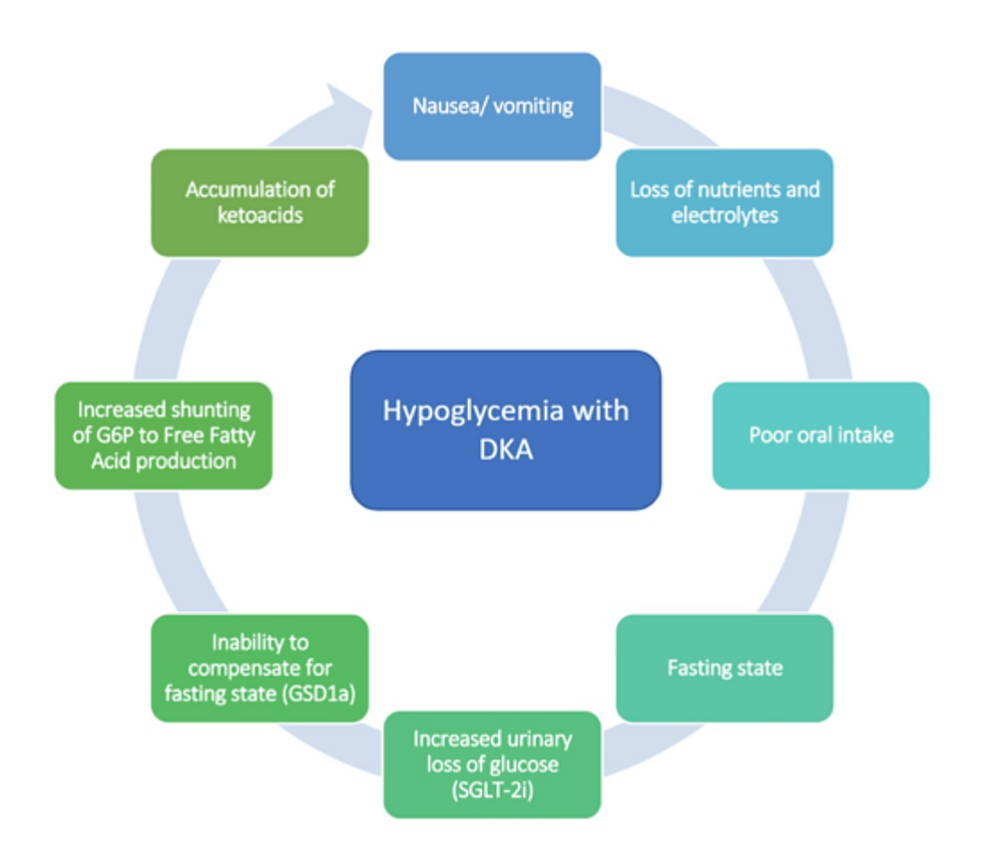
Flowchart describing the factors contributing to hypoglycemia and DKA in the patient DKA, diabetic ketoacidosis; G6P, glucose-6-phosphate; GSD1a, glycogen storage disorder 1a; SGLT-2i, sodium-glucose co-transporter-2 inhibitor The image is composed by the authors.

DKA commonly presents as hyperglycemia with elevated anion gap metabolic acidosis. Complicating factors include concurrent poor oral intake, given symptoms, use of SGLT-2 inhibitors causing increased glucose excretion, and a genetic inability to compensate for the fasting state. Hence, the patient presented with hypoglycemia and acidosis. GSD1a causes the buildup of glucose-6-phosphate, which can be shunted to the production of free fatty acids and lactate (Figure 2), which can worsen acidosis in such individuals [2].

VGD is a hepatic disorder characterized by the deficiency of G6Pase, an enzyme used for glycogenolysis. Deficiency in the enzyme leads to impaired glycogenolysis and gluconeogenesis, which can cause metabolic acidosis. Recent literature highlights the possible relationship between VGD and metabolic syndrome, which could explain the development of DM2 in our patient [3]. Melis et al. revealed that a cohort of patients with GSD1a had a statistically significant increase in traits associated with metabolic syndrome when compared to those with GSD1b and controls. Patients with GSD1a also had evidence of greater insulin resistance [4]. It has been shown that patients with diabetes treated with SGLT2 inhibitors experience euglycemic DKA episodes more frequently when their body mass is lower and glycogen stores are decreased [3]. Research also showed that SGLT2 inhibitor enhances the release of glucagon from the pancreas, which worsens existing glucagon and insulin imbalance [5-7]. 

There is limited literature on euglycemic DKA in patients with VGD. These patients typically struggle with intermittent episodes of fasting hypoglycemia. Our case report of a patient with VGD and DM2 presenting with euglycemic DKA is rare. The presence of VGD and DM2 presents a challenge to glucose homeostasis. There are few case reports of patients with different subtypes of VGD who develop DM2 in the setting of pancreatic insufficiency, which is not entirely consistent with our patient’s presentation [8,9]. In regard to DM2 and GSD1a, there are four patients found in the literature. Three of the four patients were of pediatric age, and for all the cases described, blood sugars were reduced with weight reduction [10]. Hypoglycemia contributes to disease burden in patients with GSD1a. In our case, the patient was an adult with GD1a who later developed DM2. He was known to have fasting hypoglycemic episodes at home prior to presenting with euglycemic DKA due to empagliflozin.

In a study conducted on patients with GSD1a, it was found that continuous glucose monitoring (CGM) was efficacious and reliable in monitoring asymptomatic hypoglycemia and supervising metabolic changes in drugs associated with changes in lactate. Studies using CGM showed that more frequent hypoglycemic events in a day were associated with liver adenomas and microalbuminuria in patients with GSD1a. This could explain our patient’s presentation, given the presence of multiple hepatic adenomas on his abdomen imaging (Figure 1). CGM can potentially play a role in the surveillance and prevention of hypoglycemic episodes in patients with GSD1a and DM2 [11-13]. It is important to recognize that DM2 is a rare condition when associated with VGD. It is challenging to find a regimen that would be effective in glycemic control. Empagliflozin has been shown to reduce the incidence of neutropenia and inflammatory bowel disease in VGD by inhibiting the accumulation of 1,5-anhydroglucitol-6-phosphate in leukocytes [14,15].

This patient was also found to have FAC on colon biopsies, which is a term that is descriptive of acute, patchy cryptitis that is interspersed between areas of normal colonic mucosa. The causes of FAC are varied and, per one study, were found to include drugs, mainly NSAIDs, in 24% of the individuals, infection in 19%, and chronic inflammatory bowel disease, mainly Crohn’s disease, in 16% of the individuals studied [16]. SGLT-2 inhibitors have also been used in combination with alpha-glucosidase inhibitors for the control of hyperglycemia, as studies have shown that patients with GSD1 have a delayed post-prandial response to insulin [3]. This certainly emphasizes the potential benefit of CGM to prevent episodes of hypoglycemia.

## Conclusions

Euglycemic DKA is commonly thought to occur in the event of normal blood glucose. However, it is important to recognize that VGD patients may present with hypoglycemia and elevated anion gap metabolic acidosis if they are concurrently taking an SGLT-2 inhibitor. In patients with VGD, metformin and IV fluids containing lactate can significantly worsen metabolic acidosis. SGLT-2 inhibitors, particularly empagliflozin, have been shown to reduce the incidence of neutropenia and inflammatory bowel disease in patients with VGD in addition to their antihyperglycemic properties. CGM can be used as a diagnostic modality to detect and prevent hypoglycemic events in patients with VGD.
